# Supplementary material for: An integrated multi-omic approach for discovery and dereplication of bioactive microbial natural products
Source: Microb Genom. 2025 Oct 28;11(10):001552. doi: 10.1099/mgen.0.001552 (PMC12560952; doi:10.1099/mgen.0.001552)
Supplement: Uncited Supplementary Material 1. [file mgen-11-01552-s001.pdf]

Supplementary data for An integrated multi-omic approach for discovery and dereplication of bioactive microbial natural products

Negero Gemeda Negeri[1, 2*], Duong Duc Anh Nguyen[2*], Michael Michael[2*], Emma Kenshole[2*], Calum Walsh[2,3], Louise M. Judd[3], Liam K. R. Sharkey[2], Weiguang Zeng[2], Timothy P. Stinear[2,3], Marion Herisse[2], Chuan Huang[1], Max J. Cryle[1], Sacha J. Pidot[2]

1. Biomedicine Discovery Institute, Monash University, Clayton, Australia,
2. Department of Microbiology and Immunology at the Doherty Institute, University of Melbourne, Melbourne, Australia, 3000
3. Centre for Pathogen Genomics, Doherty Institute, University of Melbourne, Melbourne, Australia, 3000

* These authors contributed equally to this study.

Contents

Table S1. Strains used in this study
Table S2. Bioactivity and compounds identified by mass spectrometry from the 65 dereplicated isolates.
Table S3. Statistics of sequenced bacterial genomes.
Table S4. Homology between proteins encoded in actinomycin BGCs.
Fig. S1. Images of diffusion chambers used in this study.
Fig. S2. Flowchart of experimental procedures.
Fig. S3. Homology between the reference BD-12 BGC and BD-12 BGC from strain 10362.
Fig. S4. Homology between the reference elaiophylin BGC and elaiophylin BGC from strain 10362.
Fig. S5. Homology between the reference geldanamycin BGC and geldanamycin BGC from strain 10362.
References

Table S1. Strains used in this study

Strain	Features	Source/reference
Escherichia coli DH10B	F⁻ *endA1 deoR⁺ recA1 galE15 galK16 nupG rpsL Δ(lac)X74* φ80*lacZΔM15 araD139 Δ(ara,leu)7697 mcrA* Δ*(mrr-hsdRMS-mcrBC)* Str^R λ⁻	Invitrogen
Staphylococcus aureus Newman	Antibiotic susceptible *S. aureus* strain.	(1)
Staphylococcus aureus JKD6008	MRSA, vancomycin-intermediate resistance, aminoglycoside and trimethoprim resistant	(2)
Klebsiella pneumoniae NTUH-K2044	Hypervirulent *K. pneumoniae*. Resistant to ampicillin.	(3)
Acinetobacter baumannii BAA-1710	Resistant to cefazolin, cefepime, cefotaxime, ceftazidime, ceftriaxone, ciprofloxacin, gentamicin, piperacillin, piperacillin-tazobactam, tetracycline, ticarcillin, trimethoprim-sulfamethoxazole	ATCC
Enterococcus faecium AUS0233	Resistant to vancomycin, trimethoprim, tetracycline, macrolides, aminoglycosides	(4)
E. coli BPH0530	Resistant to extended spectrum beta-lactams (ESBL), aminoglycosides, tetracycline, trimethoprim, quinolones	(5)
E. coli BW25113	*E. coli* K12 derivative. F^- DE(*araD-araB*)567 *lacZ4787*(del)::*rrnB*-3 *LAM⁻ rph*-1 DE(*rhaD-rhaB*)*568 hsdR514*	(6)
E. coli BW25113 (pGDP1:*stat)*	Streptothricin resistant	(7)
E. coli BW25113 (pGDP1:*uvrA*)	Echinomycin resistant	(7)

Table S2. Bioactivity and compounds identified by mass spectrometry from the 65 dereplicated isolates

Strain #	Compounds identified by GNPS, Dereplicator and Dereplicator+	Observed antibiotic activity (SaN, EcD, MRSA, VRE, Kp, Ab, EcB)*
10260	Actinomycin D	SaN, EcD, MRSA
10202	Actinomycin D, Actinomycin X	SaN, EcD, MRSA, VRE
10207	Actinomycin D, Actinomycin X	SaN, EcD, MRSA, VRE
126	Actinomycin D, Actinomycin X, Antimycin, monactin	SaN, EcD, MRSA, VRE
10218	Actinomycin D, Actinomycin X	SaN, EcD, MRSA, VRE
10256	Actinomycin D, Actinomycin X	SaN, EcD, MRSA, VRE
10269	Actinomycin X	SaN, EcD, MRSA, VRE
221	Actinomycin D, Actinomycin X, antimycin	SaN, MRSA, VRE
242	Actinomycin D, Actinomycin X, Antimycin	SaN, MRSA, VRE
10245	Actinomycin D, Actinomycin X, manumycin	SaN, EcD, MRSA, VRE
10356	Actinomycin X, novobiocin	SaN, EcD, MRSA, VRE
10191	manumycin	SaN, EcD
10194	manumycin	SaN, EcD
10195	manumycin	SaN, EcD
10199	manumycin	SaN, EcD
10200	manumycin	SaN, EcD
10253	manumycin	SaN, EcD
10271	manumycin	SaN, EcD
10248	manumycin	SaN, EcD
10192	manumycin	SaN
10193	manumycin	SaN, EcD
10247	manumycin	SaN, EcD
10251	manumycin	SaN
10252	manumycin	SaN
313	monactin	SaN, EcD
10174	monactin	SaN, EcD
202	montanastatin, valinomycin	SaN, EcD, MRSA, VRE, Kp, Ab
10125	sevadicin	SaN, EcD
143	valinomycin	EcD, Ab, Kp
10362	Elaiophylin, geldanamycin, BD-12	SaN, EcD, MRSA, VRE, EcB
326	Antimycin	SaN, EcD, MRSA, VRE
303	Antimycin	SaN
105	Antimycin	SaN, MRSA
107	Antimycin	SaN, MRSA
109	Antimycin	SaN, MRSA

118	Antimycin	SaN, MRSA
144	Antimycin	SaN
146	Antimycin	SaN, MRSA
305	Antimycin	SaN
218	Bonactin, monactin	SaN, VRE
103	Bonactin, monactin, montanastatin, valinomycin	SaN, MRSA, VRE
136	Bonactin, monactin, montanastatin, valinomycin	SaN, MRSA, VRE
201	Bonactin, monactin, montanastatin, valinomycin	SaN, MRSA, VRE
212	Bonactin, monactin, montanastatin, valinomycin	SaN, MRSA, VRE
215	Bonactin, monactin, montanastatin, valinomycin	SaN, MRSA, VRE
122	echinomycin, tambromycin A and B	SaN, MRSA, VRE
111	monactin	SaN, MRSA, VRE
125	monactin	SaN, MRSA, VRE
135	monactin	SaN, MRSA, VRE
137	montanastatin, valinomycin	SaN, MRSA
10348	novobiocin	SaN, EcD, VRE
209	pyrromycin	SaN, MRSA, VRE
210	pyrromycin	SaN, MRSA, VRE
213	Pyrromycin	SaN, MRSA, VRE
245	Pyrromycin	SaN, MRSA, VRE
203	pyrromycin, valinomycin	SaN, MRSA, VRE
217	pyrromycin, valinomycin	SaN, MRSA, VRE
133	tambromycin A and B	SaN, MRSA
106	tambromycin A and B	SaN, VRE
114	tambromycin A and B	SaN, VRE
222	tambromycin B	SaN
104	valinomycin	SaN, MRSA
204	valinomycin	SaN, MRSA
216	valinomycin	SaN, MRSA, VRE
10285	venturicidin	SaN

* If a strain is listed in this column, it means there was a discernible zone of clearing against that particular test isolate. Sa = *S. aureus* Newman; EcD = *E. coli* DH10B; MRSA = *S. aureus* JKD6008; VRE = *E. faecium* AUS0233; Kp = *K. pneumoniae* NTUH-K2044; Ab = *A. baumannii* BAA-1710; EcB = *E. coli* BPH0530. Refer to Table S1 for further information about each test organism.

Table S3. Statistics of sequenced bacterial genomes.

Strain #	Closest 16S match (% query coverage, % nt ID)	Total length	# contigs	Largest contig	Contig N50	# predicted SMBGC regions[1]	Genome assembly accession[2]	SRA accession[2]
10260	*Streptomyces murinus* NBRC 100773 (97, 100%)	8,508,414	76	569,700	357,808	36	JBPYUT000000000	SRX30805339
M126	*Streptomyces antibioticus* CSSP528 (98, 100)	9,075,813	196	360,003	97,734	32	JBPYUS000000000	SRX30805338
10202	*Streptomyces murinus* NBRC 100773 (97, 100%)	8,454,924	163	386,948	166,043	40	JBPYUR000000000	SRX30805340
10256	*Streptomyces murinus* NBRC 100773 (97, 100%)	8,718,858	2	8,395,928	8,395,928	45	JBPYUQ000000000	SRX30805341
10362	*Streptomyces antimycoticus* NBRC 100767 (97, 99.93)	11,796,174	32	2,605,409	1,130,587	52	JBPAYA000000000	SRX30805342
10258	*Streptomyces ardesiacus* (97, 99.26)	8,668,535	4	8,482,255	8,482,255	29	JBPYUP000000000	SRX30805330

[1] Indicates number of genomic regions with likely secondary metabolite BGCs (SMBGCs) as identified by AntiSMASH v7 (8). Due to fragmentation of some genome assemblies and the possibility that genes that form part of a single BGC may be present on multiple contigs, the number of SMBGCs may be artificially inflated for some genomes. The smaller the number of contigs, the more likely this number is to be an accurate reflection of secondary metabolic capacity of an individual organism. [2] Genome assemblies and read data sets can be accessed at NCBI via the listed accession numbers.

Table S4. Homology between proteins encoded in actinomycin BGCs.

Actinomycin BGC gene name	NCBI accession	Function	Orthologue locus tag in 10260	% aa ID of 10260 orthologue*	Orthologue locus tag in M126	% aa ID of M126 orthologue*	Orthologue in 10202 (% aa ID to ref prot)	% aa ID of 10202 orthologue*	Orthologue in 10256	% aa ID of 10256 orthologue*
acmrC	ADG27345.1	ATP-binding transporter	10260_05753	86	M126_03691	87	10202_00833	86	10256_00813	86
acmrB	ADG27346.1	ABC type 2 transporter	10260_05754	89	M126_03690	88	10202_00834	89	10256_00814	89
acmrA	ADG27347.1	ATP-binding transporter	10260_05755	83	M126_03689	86	10202_00835	83	10256_00815	83
acmQ	ADG27348.1	ViuB-like protein	10260_05756	77	M126_03688	82	10202_00836	77	10256_00816	77
acmP	ADG27349.1	TetR family transcriptional regulator	10260_05757	71	M126_03687	78	10202_00837	72	10256_00817	72
acmO	ADG27350.1	LbmU-like protein	10260_05758	7	M126_03686	76	10202_00838	70	10256_00818	70
acmN	ADG27351.1	ferredoxin	10260_05760	88	M126_03683	88	10202_00844	88	10256_00826	88
acmM	ADG27352.1	cytochrome P450 monooxygenase	10260_05761	59	M126_03682	60	10202_00845	60	10256_00827	59
acmL	ADG27353.1	methyltransferase	10260_05764	45	M126_03672	74	10202_00848	45	10256_00830	45
acmK	ADG27353.1	aminotransferase class V	10260_05771	73	M126_03679	45	10202_00855	73	10256_00837	73
acmT	ADG27354.1	hypothetical protein	10260_05770	74	M126_03673	72	10202_00854	74	10256_00836	74
acmS	ADG27355.1	hypothetical protein	10260_05769	81	M126_03674	80	10202_00853	81	10256_00835	83
acmR	ADG27356.1	mbtH-like protein	10260_05768	72	M126_03675	72	10202_00852	73	10256_00834	72
acmD	ADG27357.1	4-MHA carrier protein	10260_05767	72	M126_03676	73	10202_00851	72	10256_00833	73
acmA	ADG27358.1	AMP-dependent synthetase and ligase	10260_05766	73	M126_03677	72	10202_00850	73	10256_00832	73
acmB	ADG27359.1	non-ribosomal peptide synthetase	10260_05765	76	M126_03678	76	10202_00849	76	10256_00831	75
acmC	ADG27360.1	non-ribosomal peptide synthetase	10260_05764	71	M126_03672	44	10202_00848	71	10256_00830	71
acmE	ADG27360.1	hypothetical protein	10260_05771	45	M126_03679	71	10202_00855	44	10256_00837	45
acmF	ADG27361.1	aryl formamidase	10260_05763	75	M126_03680	77	10202_00847	74	10256_00829	75
acmG	ADG27362.1	tryptophan 2,3-dioxygenase	10260_05762	74	M126_03681	75	10202_00846	74	10256_00828	74
acmH	ADG27363.1	aminotransferase class V	10260_05761	84	M126_03682	82	10202_00845	85	10256_00827	84
acmI	ADG27364.1	methyltransferase	10260_05760	8	M126_03683	82	10202_00844	80	10256_00826	81
acmJ	ADG27365.1	LbmU-like protein	10260_05758	66	M126_03686	71	10202_00838	66	10256_00818	66
acmU	ADG27366.1	TetR family transcriptional regulator	10260_05757	65	M126_03687	65	10202_00837	65	10256_00817	65
acmV	ADG27367.1	ViuB-like protein	10260_05756	76	M126_03688	77	10202_00836	76	10256_00816	76
acmW	ADG27368.1	ATP-binding transporter	10260_05755	83	M126_03689	82	10202_00835	83	10256_00815	83
acmX	ADG27369.1	ABC type 2 transporter	10260_05754	84	M126_03690	85	10202_00834	84	10256_00814	84

* Percentage amino acid identity of the orthologue to the *act* BGC reference protein.

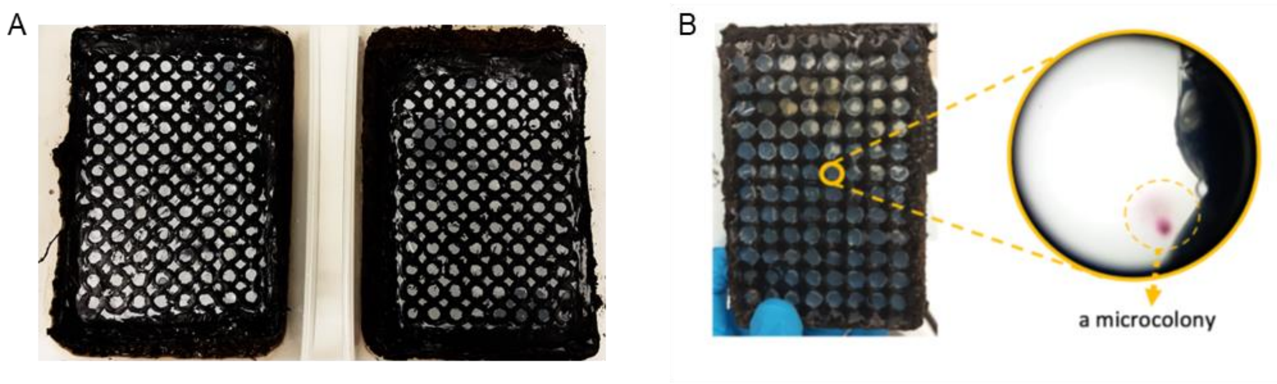

Fig. S1. Diffusion chambers as used in this study. A) Image showing constructed diffusion chambers *in situ* in their respective soil samples. B) Bottom view of a diffusion chamber following incubation. Note microbial growth can be seen in some wells as colourisation of the agar matrix. Inset shows a microcolony in an individual well as observed under a stereo microscope.

Experimental flow chart

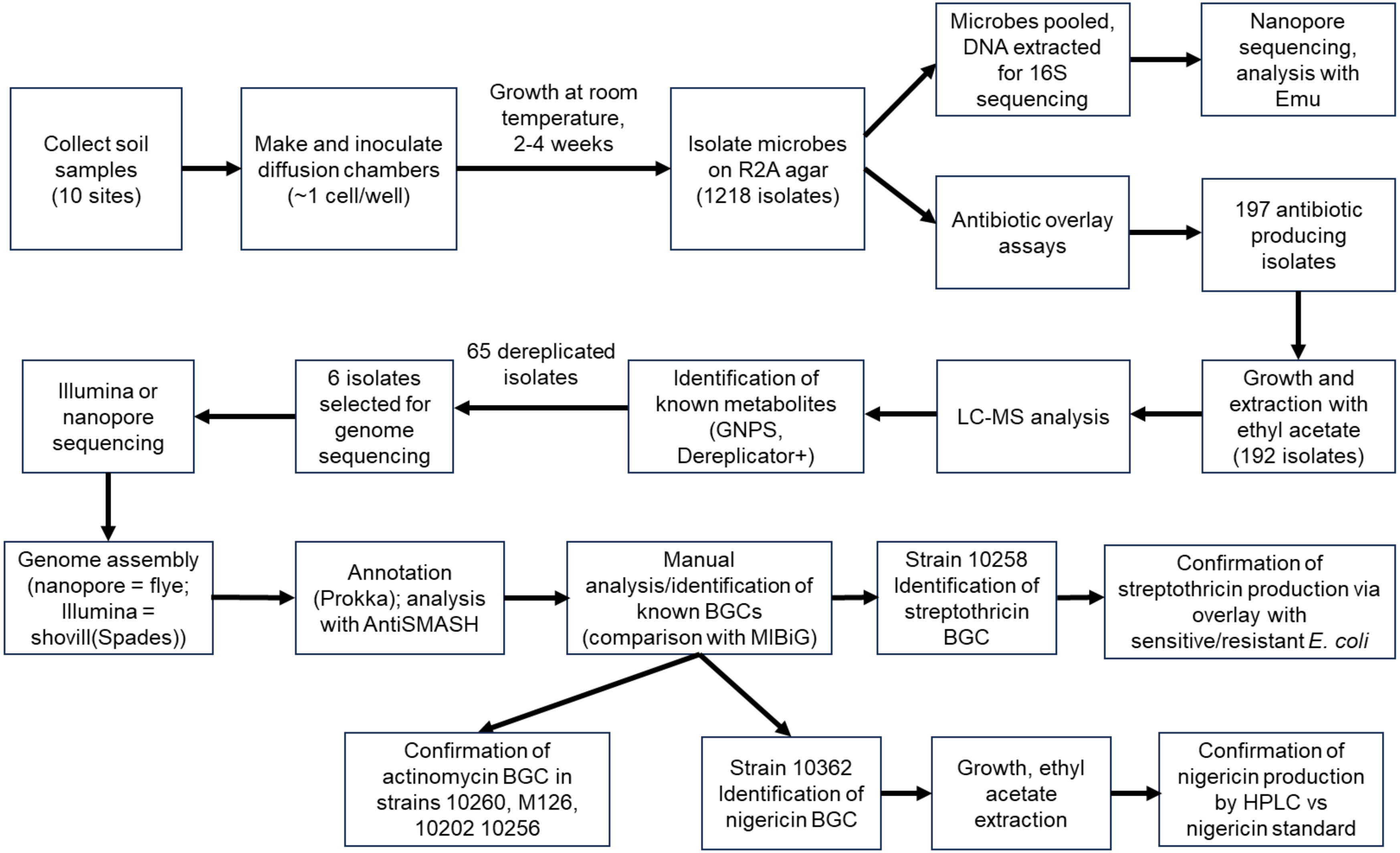

Fig. S2. Flowchart showing experimental procedures used in this manuscript.

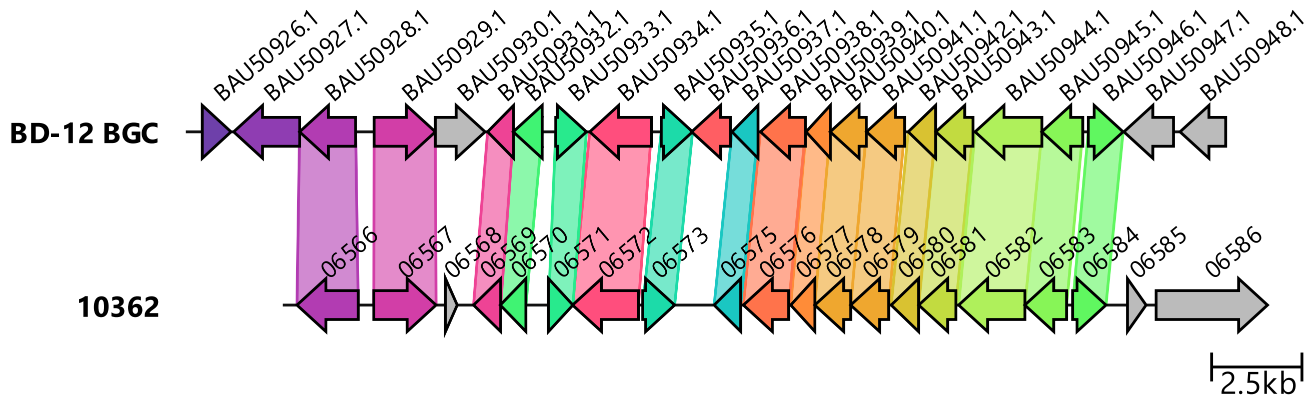

Fig. S3. Homology between the reference BD-12 BGC (MiBIG accession BGC0001379) (9) and BD-12 BGC from strain 10362.

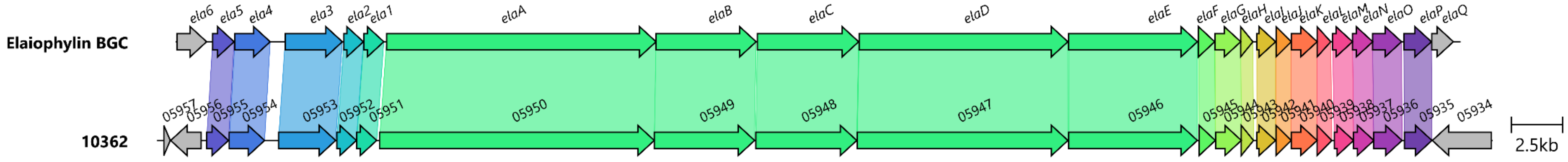

Fig. S4. Homology between the reference elaiophylin BGC (MiBIG accession BGC BGC0000053) (10) and elaiophylin BGC from strain 10362.

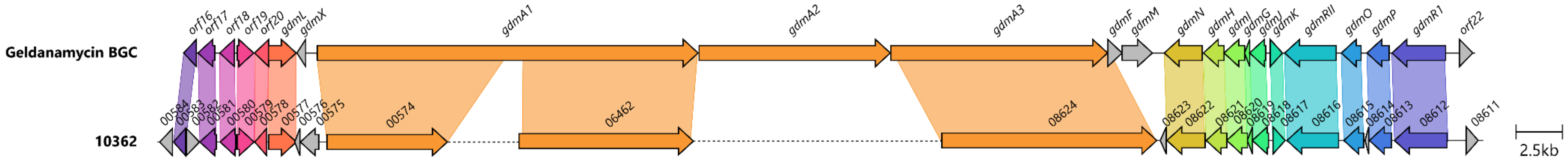

Fig. S5. Homology between the reference geldanamycin BGC (MiBIG accession BGC0000066)(11) and geldanamycin BGC from strain 10362. The dotted line in the 10362 BGC indicates a contig break.

References

1. Duthie ES, Lorenz LL. 1952. Staphylococcal Coagulase: Mode of Action and Antigenicity. Microbiology 6:95–107.

2. Howden BP, Seemann T, Harrison PF, McEvoy CR, Stanton J-AL, Rand CJ, Mason CW, Jensen SO, Firth N, Davies JK, Johnson PDR, Stinear TP. 2010. Complete Genome Sequence of Staphylococcus aureus Strain JKD6008, an ST239 Clone of Methicillin-Resistant Staphylococcus aureus with Intermediate-Level Vancomycin Resistance. J Bact 192:5848–5849.

3. Fang C-T, Chuang Y-P, Shun C-T, Chang S-C, Wang J-T. 2004. A Novel Virulence Gene in Klebsiella pneumoniae Strains Causing Primary Liver Abscess and Septic Metastatic Complications. J Exp Med 199:697–705.

4. Buultjens AH, Lam MMC, Ballard S, Monk IR, Mahony AA, Grabsch EA, Grayson ML, Pang S, Coombs GW, Robinson JO, Seemann T, Johnson PDR, Howden BP, Stinear TP. 2017. Evolutionary origins of the emergent ST796 clone of vancomycin resistant Enterococcus faecium. PeerJ 5:e2916.

5. Sherry NL, Porter JL, Seemann T, Watkins A, Stinear TP, Howden BP. 2013. Outbreak Investigation Using High-Throughput Genome Sequencing within a Diagnostic Microbiology Laboratory. J Clin Microbiol 51:1396–1401.

6. Datsenko KA, Wanner BL. 2000. One-step inactivation of chromosomal genes in Escherichia coli K-12 using PCR products. Proc Natl Acad Sci USA 97:6640–6645.

7. Cox G, Sieron A, King AM, De Pascale G, Pawlowski AC, Koteva K, Wright GD. 2017. A Common Platform for Antibiotic Dereplication and Adjuvant Discovery. Cell Chem Biol 24:98–109.

8. Blin K, Shaw S, Augustijn HE, Reitz ZL, Biermann F, Alanjary M, Fetter A, Terlouw BR, Metcalf WW, Helfrich EJN, van Wezel GP, Medema MH, Weber T. 2023. antiSMASH 7.0: new and improved predictions for detection, regulation, chemical structures and visualisation. Nucl Acids Res 51:W46–W50.

9. Maruyama C, Niikura H, Izumikawa M, Hashimoto J, Shin-ya K, Komatsu M, Ikeda H, Kuroda M, Sekizuka T, Ishikawa J, Hamano Y. 2016. tRNA-Dependent Aminoacylation of an Amino Sugar Intermediate in the Biosynthesis of a Streptothricin-Related Antibiotic. Appl Environ Microbiol 82:3640–3648.

10. Klassen JL, Lee SR, Poulsen M, Beemelmanns C, Kim KH. 2019. Efomycins K and L From a Termite-Associated Streptomyces sp. M56 and Their Putative Biosynthetic Origin. Front Microbiol 10:1739.

11. Rascher A, Hu Z, Buchanan GO, Reid R, Hutchinson CR. 2005. Insights into the Biosynthesis of the Benzoquinone Ansamycins Geldanamycin and Herbimycin, Obtained by Gene Sequencing and Disruption. Appl Environ Microbiol 71:4862–4871.
